# High-intensity treatment is common at the end of life among children and young people who died of cancer: a retrospective cohort study

**DOI:** 10.1136/bmjpo-2026-004575

**Published:** 2026-09-01

**Authors:** Stuart Jarvis, Pandell Damun, Sebastian Hinde, Helen Weatherly, Richard G Feltbower, Gayathri Subramanian, Bob Phillips, Lorna K Fraser

**Affiliations:** 1 Department of Health Sciences, University of York, York, UK; 2 Centre for Health Economics, University of York, York, UK; 3 Child Health Outcomes Research at Leeds (CHORAL), Leeds Institute for Data Analytics, University of Leeds School of Medicine, Leeds, UK; 4 Manchester University NHS Foundation Trust, Manchester, UK; 5 Centre for Reviews and Dissemination, University of York, York, UK; 6 Hull-York Medical School, University of York, York, UK; 7 Cicely Saunders Institute, King’s College London, London, UK

**Keywords:** Child Health, Palliative Care, Epidemiology

## Abstract

**Background:**

Annually, approximately 500 children and young people (aged <25 years) with cancer die in England. Little is known about the intensity of their end-of-life care.

**Aim:**

The primary aim of this study was to establish the prevalence of high-intensity treatment at the end of life in England and assess the relationship between high-intensity treatment and palliative care integration in main cancer treatment centres. The secondary aim was to assess the relationship between palliative care integration and place of death.

**Design:**

Retrospective, national full-population cohort study.

**Setting/participants:**

English Cancer Registry data with linked hospitalisation, treatment and intensive care data were used to define a cohort aged <25 years who died in 2012–2020 and post-cancer diagnosis. High-intensity treatment was defined as any intravenous chemotherapy within 14 days of death or multiple hospitalisations, emergency department visits or any intensive care unit admission within 30 days of death. High-intensity treatment prevalence at the end of life and place of death were summarised by demographics, cancer type, main treatment centre and centre-level integration of palliative care. Logistic regression models explored associations with high-intensity treatment.

**Results:**

2208 (52%) of the cohort (n=4247) had high-intensity treatments at the end of life and 1911 (45%) died in hospital. High-intensity treatment prevalence varied by age at death and cancer type. There was no evidence of health inequalities in treatments, but minority ethnic groups and those living in more deprived areas had greater odds of dying in hospital.

**Conclusions:**

High-intensity treatment is common at the end of life among children and young people with cancer; however, its appropriateness is difficult to evaluate. Nevertheless, no evidence of health inequalities was noted, and hospital deaths remain most common.

**WHAT IS ALREADY KNOWN ON THIS TOPIC**

- Around 500 children and young people with cancer die in England each year.

**WHAT THIS STUDY ADDS**

- High-intensity treatment is common at the end of life in children and young people with cancer: over half of those dying with cancer in England had high-intensity treatments.

**HOW THIS STUDY MIGHT AFFECT RESEARCH, PRACTICE OR POLICY**

- Providers should carefully assess appropriateness of high-intensity treatments and ensure a thorough discussion of care options with children and their families.

## Introduction

 Cancer is a major cause of childhood death in England,^1^ despite marked survival improvements. One in six children diagnosed with cancer in England dies, contributing nearly 500 deaths each year.^2^

Recent systematic reviews assessing the impact of specialist paediatric palliative care services for children with cancer have shown associations with low-intensity treatment at the end of life and a higher likelihood of dying outside of hospital, although evidence about quality of life and symptom management is less clear.^3–5^

Many studies have assessed end-of-life treatment intensity in adults with cancer.^6–8^ Fewer studies have focused on children, mostly in high-income countries, for example, Canada, USA and Denmark.^9–11^ An English regional study showed that specialist palliative care input was associated with fewer hospital admissions at the end of life among children with cancer,^12^ but no similar national UK study exists. This is important given the difference in health systems and funding between the UK and other high-income countries.^13^

The primary aim of this study was to establish the prevalence of high-intensity cancer treatment at the end of life among children and young people (CYP; aged <25 years) in England and assess the relationship with palliative care integration. The secondary aim was to assess the relationship between palliative care integration and place of death.

## Methods

The study was based on a published protocol^14^ and is reported according to the Strengthening the Reporting of Observational Studies in Epidemiology REporting of studies Conducted using Observational Routinely-collected health Data (STROBE RECORD) guidelines.^15^

### Study design

Retrospective, national full population cohort study

### Study population

CYP in England who met the following criteria:

1. Were diagnosed with cancer and registered to the National Cancer Registration and Analysis Service (NCRAS) aged <25 years and before 1 January 2021. Cancer diagnoses could have occurred between 1 January 1988 and 31 December 2020.
2. Died at age <25 years and before 1 January 2021, with death registered and included in Office for National Statistics (ONS) data.
3. Had not exercised a national data opt-out.

### Data sources

- NCRAS, comprising the cancer register,^16^ Systemic Anti-Cancer Therapy (SACT) dataset^17^ and Radiotherapy Dataset (RTDS).^18^
- Hospital Episode Statistics (HES), comprising admitted patient care (APC), outpatient and emergency department (ED; accident and emergency (A&E)) datasets.^19^
- Death registration records (date of death) were included in NCRAS using ONS data.^16^
- Paediatric intensive care unit (ICU) data (Paediatric Intensive Care Audit Network (PICANet)).
- Adult ICU data (Intensive Care National Audit and Research Centre (ICNARC)).

**Figure 1:**
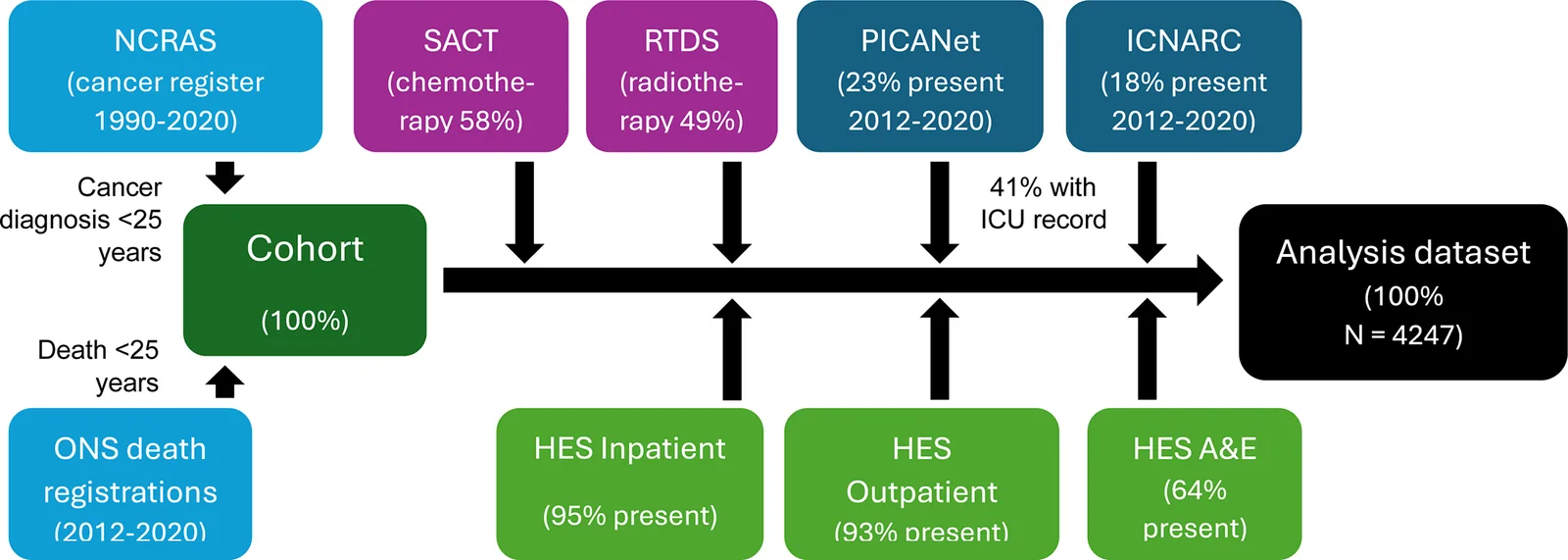
Cohort construction and datasets used. % refers to the share of the cohort present in each data source. A&E, accident and emergency; HES, Hospital Episode Statistics; ICNARC, Intensive Care National Audit and Research Centre; NCRAS, National Cancer Registration and Analysis Service; ONS, Office for National Statistics; PICANet, Paediatric Intensive Care Audit Network; SACT, Systemic Anti-Cancer Therapy.

The cohort was constructed using the cancer register and death data, with data supplemented by the other datasets (figure 1).

Approvals were obtained from a Research Ethics Committee (Ref. 21/NW/0009) and Health Research Authority Confidentiality Advisory Group (Ref. 21/CAG/0026).

### Data linkage and management

Intensive care data were linked by NHS England using deterministic data linkage on dataset serial number, NHS number, name, date of birth, sex and postcode (more details in online supplemental appendix). Data across datasets were then combined using a unique person identifier provided by NHS England.

#### Demographic data

Key demographic variables derived were as follows:

Sex, year of death and diagnoses were determined from NCRAS.

Age at death (from death registration data) and at diagnosis (from NCRAS) were categorised into five groups (under 1, 1–4, 5–9, 10–17 and 18–24 years).

Time from diagnoses to death was calculated.

Ethnic group was reduced from the 19 census categories to four owing to the low frequency: white, South Asian (Bangladeshi, Indian or Pakistani), black and mixed/other.

NCRAS included five deprivation categories based on the English Index of Multiple Deprivation (IMD) 2019.^20^ IMD is an area-based measure calculated for each lower-layer super output area, geographical areas typically comprising approximately 1500 people.

#### Clinical data

##### Cancer type

Cancer types, mapped to 12 International Classification of Childhood Cancer categories,^21^ were determined from NCRAS and reduced to three main groups for analyses: central nervous system, non–central nervous system solid tumours and haematological.

##### Main treatment centre

Main treatment centres (‘primary treatment centres’ in paediatric care; or ‘designated centres’ in teenage and young adult care) were assigned based on each cohort member’s most frequently recorded three-letter organisation data service code in NCRAS.^22^ Where no recognised main treatment centres were recorded, the most frequently recorded main treatment centre in HES APC data was used. Paediatric and TYA (teenage and young adult) centres that shared the same three-letter code were treated as a single centre in analyses.

##### Palliative care availability

Palliative care service availability and expertise within main treatment centres was determined from an earlier survey^23^ and a revised, simplified survey (online supplemental figure S1) to address non-response to the original survey.

Embeddedness of medical and nursing palliative care expertise and age-appropriate consult-led palliative care were categorised as absent, partial or strong, as previously reported.^23^ These were collapsed into a combined measure: 1 if there was strong embeddedness of palliative care (palliative care integration) across all three domains and 0 otherwise.

### Outcomes data

The main outcome (high-intensity treatment at end of life) was based on existing definitions^11^ and informed by online consultation with 12 UK paediatric oncology and haematology experts in June 2021.^14^ High-intensity treatment was defined as any one or more of the following:

- Intravenous chemotherapy within 14 days of death.
- More than one ED visit within 30 days of death.
- More than one hospitalisation within 30 days of death.
- Any ICU admission within 30 days of death.

Secondary outcomes were also used, based on clinician input at the consultation event:

- Mechanical ventilation within 14 days of death.
- Radiotherapy within 14/30 days of death.
- Haematopoietic stem cell transplantation (HSCT) within 100 days of death.
- Oral or intravenous chemotherapy within 14 days of death.
- Place of death

Place of death, from ONS death registration data, was categorised as hospital, hospice, private residence and other. Further details on the derivation of outcomes are provided in the online supplemental appendix.

### Patient and public involvement

Study design and use of patient-identifiable data without consent were discussed with four bereaved parents of children who had cancer, from the Martin House Research Centre Family Advisory Board (www.york.ac.uk/mhrc) and Paediatric Oncology Reference Team (PORT). PORT is a team of parents in the UK who have direct experience of children’s cancer. These parents were supportive of the use of their child’s data without consent for data linkage. They reviewed the text for the website, suggesting some changes which were made by the research team.

The study was also supported by a study-specific parent advisory panel, and the findings were discussed with them.

### Analysis

#### Descriptive analysis

Numbers of cohort members who received high-intensity treatment at the end of life (primary combined measure, components thereof and secondary outcomes) were summarised graphically and split by key clinical and demographic characteristics. Place of death was separately summarised.

#### Multivariable regression

Logistic regressions explored associations between high-intensity treatment at the end of life (outcome), integration of specialist paediatric palliative care (main exposure of interest) and other demographic and clinical variables. A directed acyclic graph (DAG, online supplemental figure S12, online supplemental appendix) set out expected causal relationships and determined appropriate adjustment variables. As the data were clustered in main treatment centres, a random intercept model was used. Secondary exposures of interest were age at death, sex, ethnic group, deprivation category, cancer category and year of death. Time to death and age at death were highly collinear, so age at death was included in the final models.

Associations between demographic and clinical variables and place of death—hospital versus non-hospital—were explored in the same way with support from a separate DAG (online supplemental figure S13, online supplemental appendix).

Analyses were repeated for those who were diagnosed before and after age 18 years. Analyses were repeated, excluding deaths from 2020 (ie, covering only deaths from 2012 to 2019) as a sensitivity analysis for possible impacts of the COVID-19 pandemic.

## Results

### Cohort characteristics

**Figure 2:**
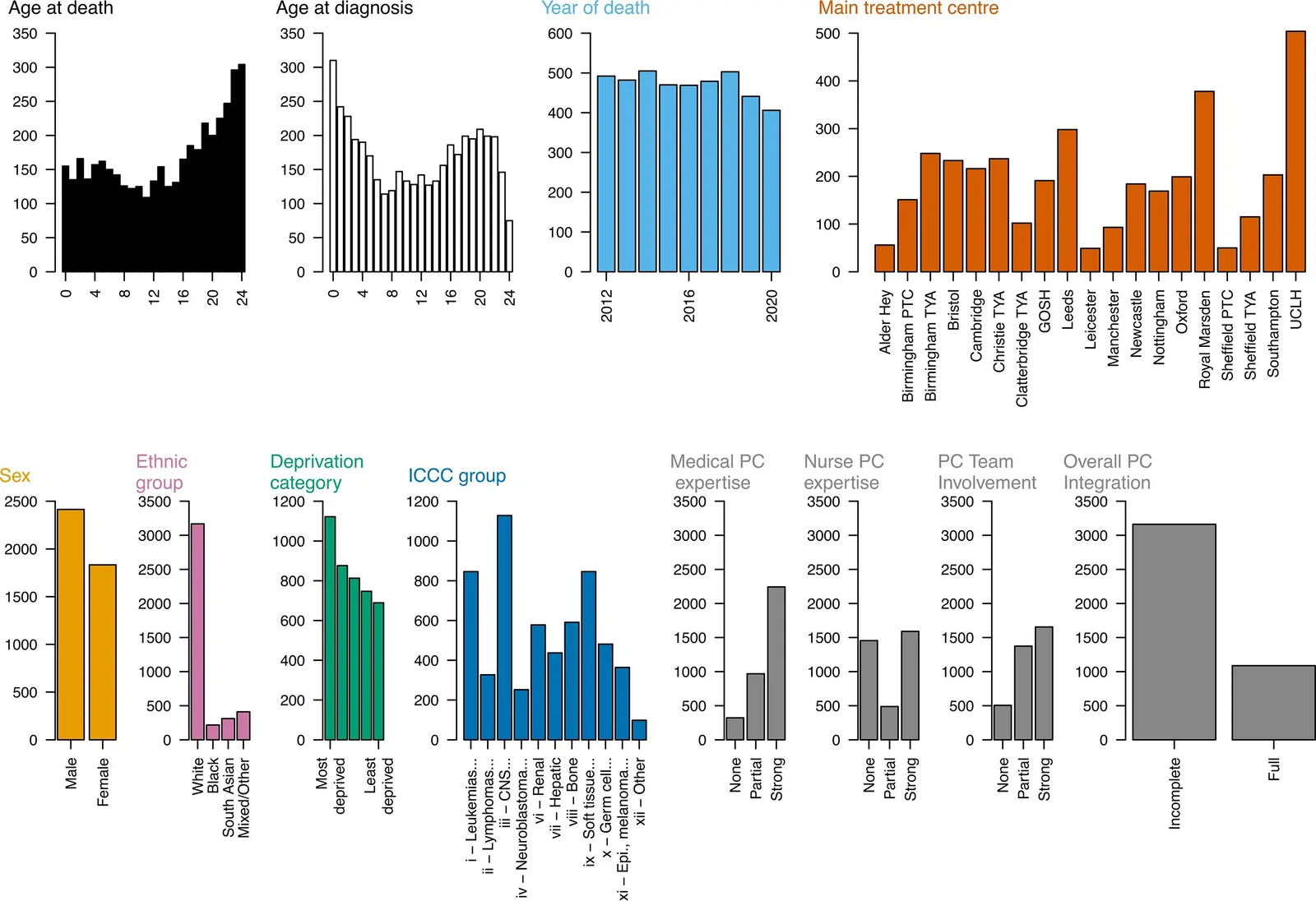
Composition of cohort. CNS, central nervous system; GOSH, Great Ormond Street Hospital; ICCC, International Classification of Childhood Cancer; PC, palliative care; PTC, Paediatric Treatment Centre; TYA, teenage and young adult; UCLH, University College London Hospitals.

The cohort included 4247 CYP (figure 2). There was a skew towards older age of death. Cancer diagnoses were mostly made in the first few years of life or later teenage years. Deaths were evenly spread across the study period (2012–2020) with a slight reduction in 2020 (possibly related to registration delays during the COVID-19 pandemic). There were more males (57%) than females, and the cohort was predominantly white (75%). There was a deprivation gradient, with more from more deprived areas. Central nervous system, solid cancers and leukaemia were the most common cancers. Very few children with retinoblastoma died; these were excluded from subsequent analyses. Numbers in main treatment centres varied widely, from 49 to 504. 356 cohort members (6%) lacked recorded treatment at a main treatment centre, mostly those dying at age <1 year or >18 years.

### Palliative care

Data on palliative care were available for 16 out of 19 main treatment centres (online supplemental appendix) covering 84% of cohort members (91% of those assigned a main treatment centre). A summary of responses is provided in online supplemental figure S2 (online supplemental appendix). All responding centres reported referrals to hospices. On the combined measure, six centres reported full integration of palliative care across all three domains before 2020.

### High-intensity treatment at the end of life

#### Descriptive summary

**Figure 3:**
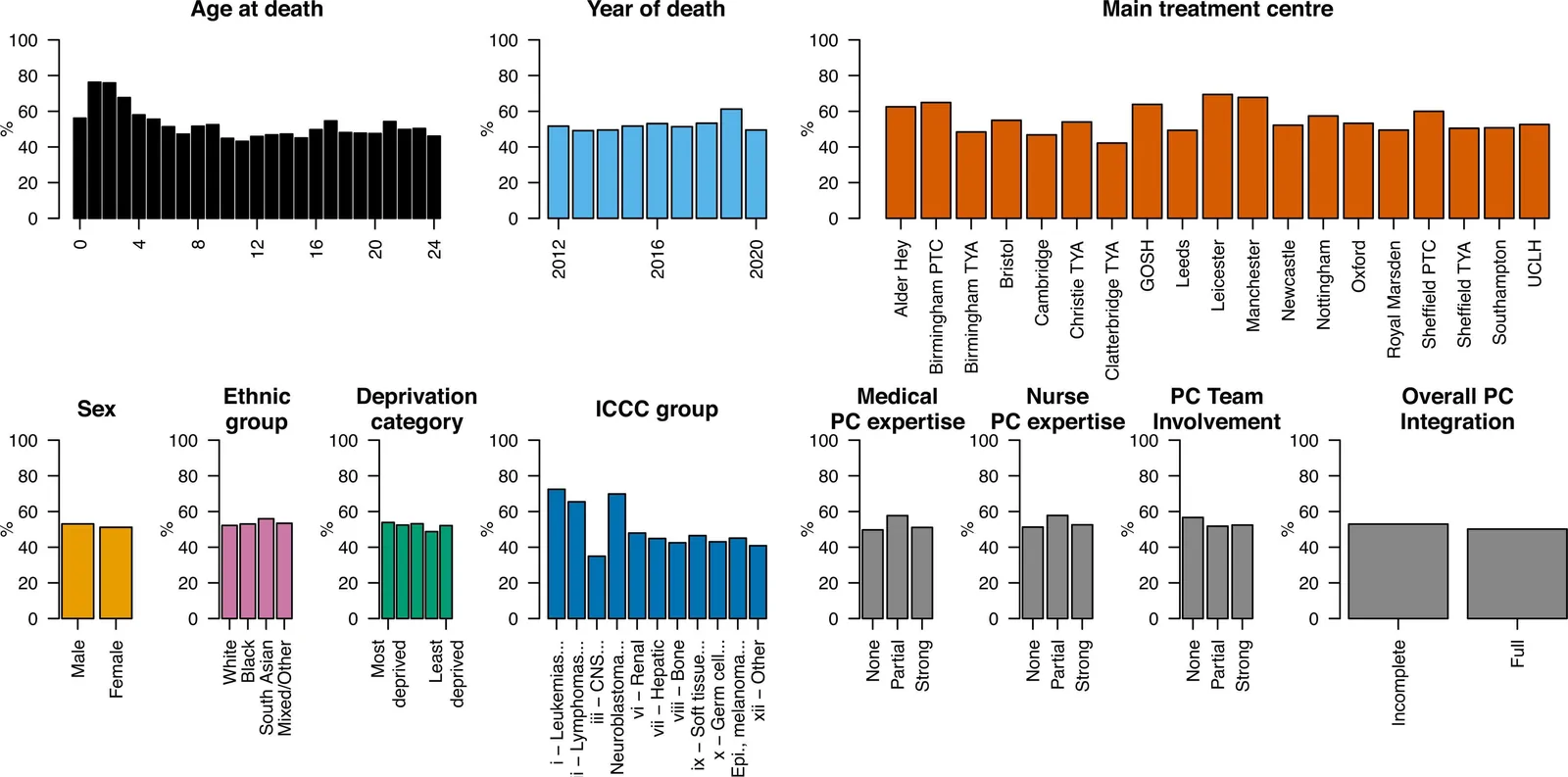
Prevalence (%) of high-intensity treatment at the end of life. CNS, central nervous system; GOSH, Great Ormond Street Hospital; ICCC, International Classification of Childhood Cancer; PC, palliative care; PTC, Paediatric Treatment Centre; TYA, teenage and young adult; UCLH, University College London Hospitals.

52% of cohort members had evidence of high-intensity treatment at the end of life. It was most common for children dying aged 1–3 years and for those with leukaemia, lymphomas or neuroblastoma (figure 3). High-intensity treatment was more common in those with shorter duration of time to death (online supplemental table S1). The sexes, ethnic groups and deprivation categories had similar prevalence of high-intensity treatment, as did those in centres with or without integration of palliative care services. A descriptive summary of variations in components of the high-intensity treatment measure and in secondary measures of high-intensity treatment is provided in online supplemental figures S3–S11.

#### Multivariable model

Logistic regression analysis showed lower (but not statistically significant) odds of high-intensity treatment for those treated in centres with full versus incomplete specialist palliative care integration (OR 0.86, 95% CI 0.74 to 1.01) (table 1). Those who died at age 1–4 years or <1 year had higher odds (respective ORs 2.33, 95% CI 1.85 to 2.93 and 1.58, 95% CI 1.07 to 2.33) than those who died at age 5–10 years. Haematological cancers were associated with higher odds of high-intensity treatment (OR 2.72, 95% CI 2.28 to 3.24) and central nervous system tumours with lower odds (OR 0.45, 95% CI 0.37 to 0.53) than those with solid tumours. The odds of high-intensity treatment increased through time (OR from 1 year to previous year 1.03, 95% CI 1.00 to 1.06). There were no differences in the likelihood of high-intensity treatment by sex, ethnic group or deprivation category.

**Table 1:** Multivariable logistic regression model results for risk of high-intensity treatment at the end of life

	Outcome: high-intensity treatment at the end of life		Outcome: hospital as a place of death	
	OR	95% CI	OR	95% CI
Specialist paediatric palliative care integration (ref: incomplete integration)*				
Full integration	0.86	0.74 to 1.01	0.65	0.44 to 0.97
Age at death (ref: 5–10 years)†				
<1	1.58	1.07 to 2.33	3.22	2.13 to 4.87
1–4	2.33	1.85 to 2.93	1.62	1.29 to 2.04
11–15	0.82	0.66 to 1.01	1.21	0.97 to 1.51
16–17	1.07	0.83 to 1.38	1.56	1.20 to 2.04
18–25	0.94	0.79 to 1.12	1.84	1.53 to 2.22
Sex (ref: male)‡				
Female	0.92	0.81 to 1.05	1.00	0.87 to 1.13
Ethnic group (ref: white)‡				
Black	1.00	0.74 to 1.35	2.32	1.70 to 3.16
South Asian	1.08	0.85 to 1.38	1.92	1.50 to 2.47
Mixed or other	1.03	0.83 to 1.28	1.75	1.40 to 2.18
Deprivation category (ref: 1, most deprived)§				
2	1.02	0.84 to 1.24	0.83	0.68 to 1.01
3	1.02	0.84 to 1.25	0.76	0.62 to 0.93
4	0.83	0.68 to 1.01	0.70	0.57 to 0.87
5, least deprived	0.99	0.81 to 1.22	0.66	0.53 to 0.82
Cancer category (ref: solid tumours)¶				
Haematological	2.72	2.28 to 3.24	3.58	3.00 to 4.26
Central nervous system	0.45	0.37 to 0.53	0.76	0.63 to 0.92
Year of death (per year)‡	1.03	1.00 to 1.06	1.02	0.99 to 1.05

* Adjusted for age at death, sex, ethnic group, deprivation category, cancer category and year of death.

† Adjusted for year of death.

‡ No adjustment needed.

§ Adjusted for age at death, sex, ethnic group and year of death.

¶ Adjusted for age at death, sex, ethnic group, deprivation category and year of death.

Results from the sensitivity analysis excluding deaths in 2020 and split by age of diagnoses (before and after age 18 years) were similar (online supplemental tables S2 and S3, online supplemental appendix).

### Place of death

#### Descriptive summary

**Figure 4:**
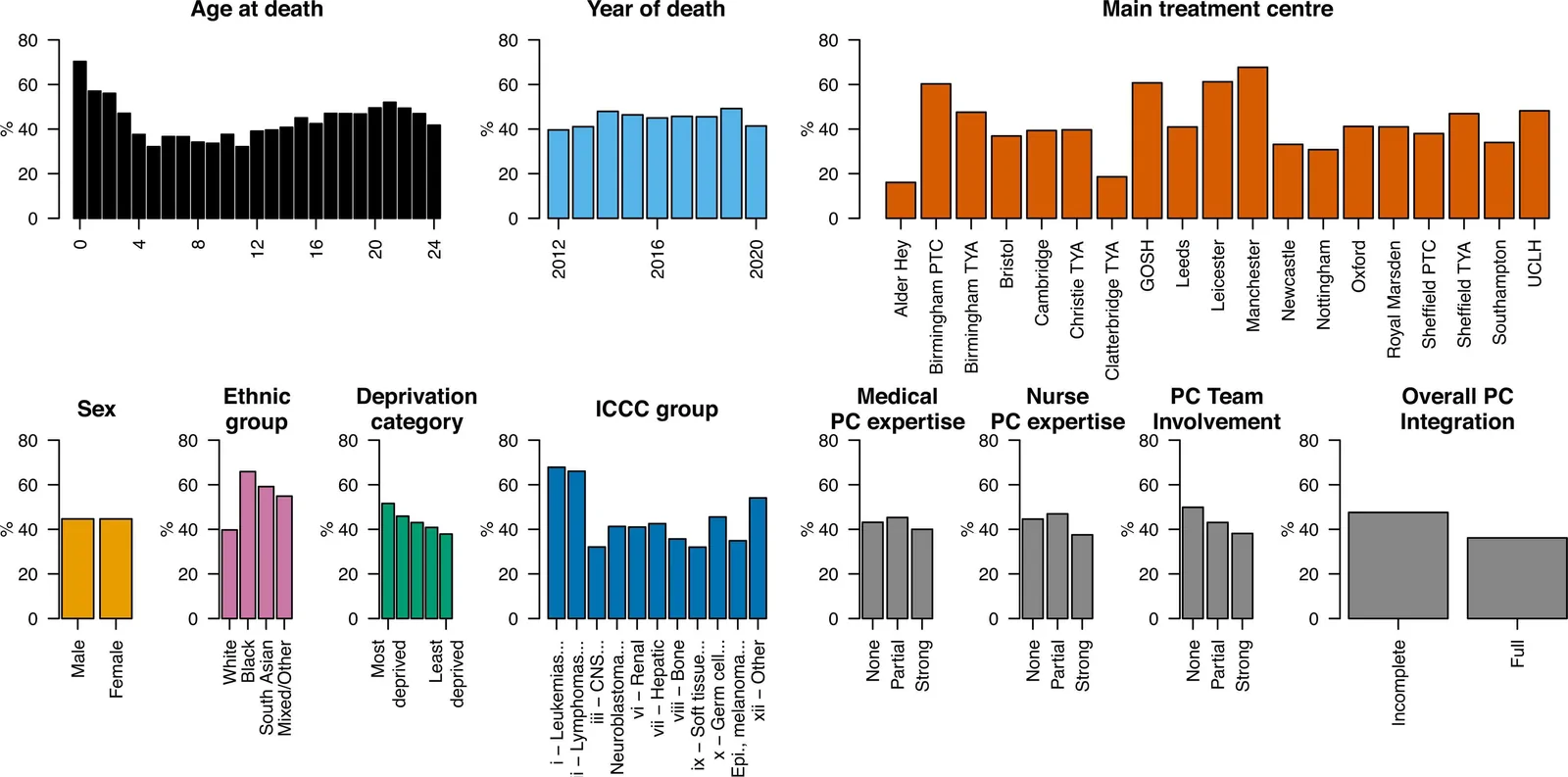
Prevalence of hospital deaths for the cohort. CNS, central nervous system; GOSH, Great Ormond Street Hospital; ICCC, International Classification of Childhood Cancer; PC, palliative care; PTC, Paediatric Treatment Centre; TYA, teenage and young adult; UCLH, University College London Hospitals.

Most children died somewhere other than hospital (home, 36%; hospice, 11%; hospital, 45%; other or unknown settings, 8%, with the vast majority of these unknown). Hospital deaths were most common for those aged <1 year (70%, figure 4), least common for those aged 5–11 years (11 years: 32%) and increased through teenage years (21 years: 52%).

Non-white groups more commonly died in hospitals as did those from more deprived areas. Hospital deaths were most common for leukaemia (68%) and lymphoma (66%) and least common for central nervous system or soft tissue cancers (both 32%). Main treatment centres varied from 16% to 68% of in-hospital deaths. Where medical or nurse palliative care expertise or palliative care team involvement was high, hospital deaths were fewer (combined measure on palliative care integration: full, 36% hospital deaths; incomplete, 48% hospital deaths).

#### Multivariable model

Centres that had full versus incomplete integration of specialist palliative care had lower odds (0.65, 95% CI 0.44 to 0.97) of being the place of death (table 1). Deaths occurring at age <1 year had higher odds (3.22, 95% CI 2.13 to 4.87) of occurring in hospitals compared with the reference group (5–10 years). Deaths occurring at age 1–4 years, or 16–25 years, also had higher odds of occurring in hospital (table 1). Those from non-white ethnic groups had higher odds of hospital death than the white ethnic group (black ethnic group, OR 2.32, 95% CI 1.70 to 3.16; South Asian, 1.92, 95% CI 1.50 to 2.47; mixed/other, 1.75, 95% CI 1.40 to 2.18). Those from less-deprived areas had lower odds of hospital death than the most deprived (least deprived group: OR 0.66, 95% CI 0.53 to 0.82). Those with haematological cancers had much higher odds of death in hospital than those with solid tumours (OR 3.58, 95% CI 3.00 to 4.26); central nervous system tumours had lower odds (OR 0.76, 95% CI 0.63 to 0.92). Sex and year of death were not associated with likelihood of hospital death.

Results from the sensitivity analysis excluding deaths in 2020 were substantially similar (online supplemental table S1, online supplemental appendix).

## Discussion

More than half of all CYP in England who died after a cancer diagnosis received high-intensity treatment at the end of life. High-intensity treatment was more likely in those with haematological malignancies and in children aged <5 years. There was no evidence of inequalities in high-intensity treatment use, nor of a clear link between high-intensity treatment and integration of specialist palliative care. A slight increase in high-intensity treatment prevalence was observed over time.

While most CYP with cancer died outside hospital, this varied: children aged <5 and >16 were more likely to die in hospital. The odds of hospital death were higher in haematological cancers, among those from minority ethnic groups and in areas of higher deprivation. Full integration of palliative care services at treatment centres was associated with a one-third reduction in hospital deaths.

This study’s 52% prevalence of high-intensity treatment is lower than those reported in the USA^9^ (65%), France^10^ (82.8%) and Denmark^24^ (74.3%) but higher than that in Canada^11^ (40.6%). Nevertheless, studies showed similar relationships for age and haematological malignancies,^9–11^ potentially reflecting differing prognoses and certainty around trajectories.

This study did not find evidence of inequalities in high-intensity treatment at the end of life, which was different from the findings of studies in the USA (which showed a higher use of high-intensity treatment in minority ethnic groups^9^) and Denmark (which showed higher high-intensity treatment with higher deprivation).^24^

In adults, high-intensity treatment at the end of life is often considered inappropriate, but interpretation for children is more complex, especially for haematological cancers where treatment-related deaths are not uncommon.^25^ This study could not determine whether deaths were treatment-related or disease-related (however, a Canadian study found that outcomes did not change when treatment-related deaths were excluded).^11^ We also had no information on choices offered to children and parents.

Integration of palliative care varied, as reported previously,^23^ and was not associated with high-intensity treatment. A Canadian study found reduced high-intensity treatment with specialist paediatric palliative care team involvement but was not associated with general palliative care.^11^ In the present study, specialist palliative care service availability was described for main treatment centres but not for individual cohort members.

The proportion of CYP with cancer dying in hospital (approximately45%) has changed slightly over the last few decades in England.^26^ This is similar to Canada^11^ (43.4%) but lower than those in the USA^9^ (63%), Denmark^24^ (69%) and Taiwan^3^ (78%). In the present study, minority groups and those from more deprived areas were more likely to die in hospital. US data also show that white children with cancer are more likely than non-white children to die at home.^27^ Although most parents prefer their child to die at home, especially in cancer cases, we have little information from parents in minority ethnic groups.^28^

Systematic reviews have shown that specialist palliative care input is associated with lower-intensity treatment at the end of life and dying outside of a hospital setting.^3–5^ This study supports the latter but not the former. However, palliative care integration was only measured at the treatment centre level; we had no information on palliative care individual cohort members received.

### Strengths and limitations

This study used a national, full population dataset but has some limitations. First, cancer treatment data may be incomplete in both NCRAS and hospital data. We did not have access to the British Society of Blood and Marrow Transplantation and Cellular Therapy Data Registry,^29^ possibly leading to under-recording of HSCT, particularly for those aged>16 years (PICANet data recorded transplants, but ICNARC data did not). Differentiation between allogenic and autogenic transplants was not possible.

Second, the data lacked information on whether treatments were palliative or curative (except for the RTDS, but here, there was uncertainty about the completeness of this information). We could not ascertain whether deaths were treatment-related and had no data on choices available to families or cohort member-level palliative care involvement. This prevented evaluation of the appropriateness of high-intensity treatment.

Third, the components of high-intensity treatment vary in intensity: care meeting the criteria for individual components could differ substantially. A transplant may be seen as far more intensive than intravenous chemotherapy, which covers a range of intensities and palliative and curative intent.

Fourth, the measure of embeddedness of palliative care relies on centre self-report and does not capture all relevant aspects of care. Limitations must be considered in assuming that responses to the earlier survey can be applied to the whole period (those who responded to the new survey specified when aspects of service began). This may lead to some misclassification and could bias the results towards the null, that is, no association between the embeddedness of palliative care and high-intensity treatment at the end of life or the place of death.

Fifth, the COVID-19 pandemic and dissolution of Public Health England, to which data applications were originally submitted, meant that the data were slightly outdated (running only to 2020) when delivered in 2024.

Finally, efforts to use binomial models with a log-link and more easily interpreted relative risks rather than ORs failed because of convergence issues, so logistic regression was used.

### Implications for research

Cancer treatment data in England are rich, but information on delivered palliative care, treatment types, curative versus palliative intent and whether deaths were treatment-related is limited. Noting that some of these data, that is, curative or palliative intent, should be completed in the existing SACT dataset but were mainly missing. Some of these other data items could be added to registries; others may require primary data collection.

The appropriateness of any high-intensity treatment at the end of life should be investigated, for example, through case reviews.

This study found evidence of inequalities in the place of death. More research is needed to understand whether this reflects differences in preferences or in what is made available (and, in both cases, why).

Timely provision of research data is essential for rapid dissemination of up-to-date findings to inform care improvements.

### Implications for practice

There was no evidence for inequalities in high-intensity treatments at the end of life in the overall measure, but descriptive summaries showed variations in ED use at the end of life. Providers should consider these differences: although some, such as apparent variations in ED use between age groups, may reflect intentionally different care models.

Given the high prevalence of high-intensity treatments at the end of life, providers should continue to carefully consider their appropriateness and ensure advanced care plans are in place, supported by a thorough discussion of care options.

While specialist palliative care integration was not clearly associated with high-intensity treatment at the end of life, it was clearly associated with greater chances of non-hospital death. Providers should provide comprehensive support to enable the preferred place of death.

## Conclusions

In this study, the proportion of children with cancer receiving high-intensity treatment at the end of life (52%) is one of the lowest published to date, but there was a small increase over time. The groups more likely to receive high-intensity treatment were children with haematological malignancies and children aged <5 years. The percentage of children dying outside of hospital is one of the highest reported worldwide; however, evidence shows inequalities in which children from minority ethnic groups and highly deprived areas are more likely to die in hospital. Palliative care integration varies across the main treatment centres, but those with full integration were associated with lower likelihood of hospital death.

## Supplementary material

10.1136/bmjpo-2026-004575 online supplemental file 1

## Data Availability

Data may be obtained from a third party and are not publicly available.

## References

[1] Office for National Statistics (ONS) (2025). Child and infant mortality in England and Wales: 2023. https://www.ons.gov.uk/peoplepopulationandcommunity/birthsdeathsandmarriages/deaths/bulletins/childhoodinfantandperinatalmortalityinenglandandwales/2023.

[2] The Children & Young People’s Cancer Association (2025). Children and young people’s cancer in numbers. https://www.cclg.org.uk/about-cancer/cancer-children-and-young-people/children-and-young-peoples-cancer-numbers.

[3] Lin S-C, Huang M-C, Yasmara D (2021). Impact of palliative care on end-of-life care and place of death in children, adolescents, and young adults with life-limiting conditions: A systematic review. Palliat Support Care.

[4] Taylor J, Booth A, Beresford B (2020). Specialist paediatric palliative care for children and young people with cancer: A mixed-methods systematic review. Palliat Med.

[5] Kaye EC, Weaver MS, DeWitt LH (2021). The Impact of Specialty Palliative Care in Pediatric Oncology: A Systematic Review. J Pain Symptom Manage.

[6] Lawday S, Zucker BE, Gardner S (2024). Surgical Treatment Intensity at the End of Life in Patients With Cancer: A Systematic Review. Ann Surg Open.

[7] Ma Z, Li H, Zhang Y (2024). Prevalence of aggressive care among patients with cancer near the end of life: a systematic review and meta-analysis. EClinicalMedicine.

[8] Abedini NC, Hechtman RK, Singh AD (2019). Interventions to reduce aggressive care at end of life among patients with cancer: a systematic review. Lancet Oncol.

[9] Johnston EE, Alvarez E, Saynina O (2017). Disparities in the Intensity of End-of-Life Care for Children With Cancer. Pediatrics.

[10] Revon-Rivière G, Pauly V, Baumstarck K (2019). High-intensity end-of-life care among children, adolescents, and young adults with cancer who die in the hospital: A population-based study from the French national hospital database. Cancer.

[11] Kassam A, Sutradhar R, Widger K (2017). Predictors of and Trends in High-Intensity End-of-Life Care Among Children With Cancer: A Population-Based Study Using Health Services Data. J Clin Oncol.

[12] Fraser LK, van Laar M, Miller M (2013). Does referral to specialist paediatric palliative care services reduce hospital admissions in oncology patients at the end of life?. Br J Cancer.

[13] Wolfe I (2015). Child health, health services and systems in UK and other European countries.

[14] Papworth A, Hackett J, Beresford B (2022). End of life care for infants, children and young people (ENHANCE): Protocol for a mixed methods evaluation of current practice in the United Kingdom [version 1; peer review: 2 approved]. NIHR Open Res.

[15] Benchimol EI, Smeeth L, Guttmann A (2015). The REporting of studies Conducted using Observational Routinely-collected health Data (RECORD) statement. PLoS Med.

[16] Henson KE, Elliss-Brookes L, Coupland VH (2020). Data Resource Profile: National Cancer Registration Dataset in England. Int J Epidemiol.

[17] Pickering L, Larkin J (2014). Systemic anti-cancer therapy (SACT) dataset. Lancet Oncol.

[18] Sandhu S, Sharpe M, Findlay Ú (2023). Cohort profile: radiotherapy dataset (RTDS) in England. BMJ Open.

[19] Herbert A, Wijlaars L, Zylbersztejn A (2017). Data Resource Profile: Hospital Episode Statistics Admitted Patient Care (HES APC). Int J Epidemiol.

[20] Bowie P (2019). The English indices of deprivation 2019—statistical release.

[21] Steliarova-Foucher E, Stiller C, Lacour B (2005). International Classification of Childhood Cancer, third edition. Cancer.

[22] NHS England (2025). ODS data search and export. https://www.odsdatasearchandexport.nhs.uk/.

[23] Bedendo A, Papworth A, Beresford B (2025). End of life care in paediatric settings: UK national survey. BMJ Support Palliat Care.

[24] Wolff S, Christiansen CF, Johnsen SP (2021). Disparities in intensity of treatment at end-of-life among children according to the underlying cause of death. Acta Paediatr.

[25] Hassan H, Rompola M, Glaser AW (2017). Validation of a classification system for treatment-related mortality in children with cancer. BMJ Paediatr Open.

[26] Gao W, Verne J, Peacock J (2016). Place of death in children and young people with cancer and implications for end of life care: a population-based study in England, 1993-2014. BMC Cancer.

[27] Cawkwell PB, Gardner SL, Weitzman M (2015). Persistent racial and ethnic differences in location of death for children with cancer. Pediatr Blood Cancer.

[28] Bluebond-Langner M, Beecham E, Candy B (2013). Preferred place of death for children and young people with life-limiting and life-threatening conditions: a systematic review of the literature and recommendations for future inquiry and policy. Palliat Med.

[29] British Society of Blood and Marrow Transplantation and Cellular Therapy (2025). BSBMTCT data registry. https://bsbmtct.org/about-the-registry/%20(2025.

