# Supplementary material for: High-intensity treatment is common at the end of life among children and young people who died of cancer: a retrospective cohort study
Source: BMJ Paediatr Open. 2026 Sep 1;10(1):e004575. doi: 10.1136/bmjpo-2026-004575 (PMC13536170; doi:10.1136/bmjpo-2026-004575)
Supplement: Supplementary file 1 [file bmjpo-2026-004575.reviewer_comments.pdf]

PEER REVIEW HISTORY

BMJ Paediatrics Open publishes all reviews undertaken for accepted manuscripts. Reviewers are asked to complete a checklist review form and are provided with free text boxes to elaborate on their assessment. These free text comments are reproduced below.

ARTICLE DETAILS

**TITLE (PROVISIONAL)**	High intensity treatment is common at end-of-life for children and young people who died with cancer: a retrospective cohort study
**AUTHORS**	Jarvis, Stuart<br>Damun, Pandell<br>Hinde, Sebastian<br>Weatherly, Helen<br>Feltbower, Richard G<br>Subramanian, Gayathri<br>Phillips, Bob<br>Fraser, Lorna K

VERSION 1 – REVIEW

**REVIEWER NAME**	Reviewer 1
**REVIEWER AFFILIATION**	
**REVIEWER CONFLICT OF INTEREST**	No Conflict of Interest
**AI DISCLOSURE**	No, I have not used AI
**DATE REVIEW RETURNED**	19-Dec-2025

**GENERAL COMMENTS**	Comments not published pending reviewer's consent

**REVIEWER NAME**	Reviewer 2
**REVIEWER AFFILIATION**	
**REVIEWER CONFLICT OF INTEREST**	No Conflict of Interest
**AI DISCLOSURE**	No, I have not used AI
**DATE REVIEW RETURNED**	

**GENERAL COMMENTS**	Comments not published pending reviewer's consent

**REVIEWER NAME**	Reviewer 3
**REVIEWER AFFILIATION**	
**REVIEWER CONFLICT OF INTEREST**	No Conflict of Interest
**AI DISCLOSURE**	No, I have not used AI
**DATE REVIEW RETURNED**	

**GENERAL COMMENTS**	Comments not published pending reviewer's consent

VERSION 2 – REVIEW

**REVIEWER NAME**	Yotani, Nobuyuki
**REVIEWER AFFILIATION**	Kokuritsu Kenkyu Kaihatsu Hojin Kokuritsu Seiiku Iryo Kenkyu Center, palliative medicine
**REVIEWER CONFLICT OF INTEREST**	No Conflict of Interest
**AI DISCLOSURE**	I use AI (deepl) to help make the English text easier to understand.
**DATE REVIEW RETURNED**	29-May-2026

**GENERAL COMMENTS**	General Comments: Thank you for submitting this interesting and clinically relevant study on the intensity of end-of-life care for children and young adults with cancer in England. The findings provide valuable insights into current practices and highlight important areas for service provision. However, I have a few suggestions regarding the characteristics of the cohort and the analytical approach that could strengthen the manuscript further.<br>Specific comments:<br>1. Impact of Disease Duration (Time from Diagnosis to Death): The duration from cancer diagnosis to death may significantly impact the likelihood of receiving high-intensity treatment at the end of life. For example, patients with a longer disease trajectory may have more opportunities to accept their condition and plan for end-of-life care, which could reduce the intensity of treatment near death. Conversely, rapid progression may lead to more aggressive interventions. Therefore, I strongly recommend adding descriptive data on disease duration (e.g., median time from diagnosis to death or its distribution) to the cohort characteristics, at a minimum. If the data allows, it would be highly beneficial to provide an evaluation or a stratified analysis based on disease duration or adjust for it in the multivariable model. This would provide a more nuanced understanding of end-of-life decision-making.<br>2. Heterogeneity of the Cohort (Age Cut-Off at 25 Years): I have a minor concern about the age inclusion criteria (under 25 years old). While this captures the adolescent and young adult (AYA) population, it introduces heterogeneity into the cohort. This age group includes not only typical childhood-onset cancers but also adult-type cancers, such as epithelial carcinomas, cervical cancer, and melanomas. The biological behavior, progression speed, and end-of-life decision-making processes may differ substantially between childhood-type and adult-type cancers, as the latter are often driven by patients themselves rather than solely by parents, as is the case with older individuals. Could the authors comment on how this mixture might affect the overall cohort characteristics? A brief discussion of this limitation or, ideally, a supplementary analysis separating typical pediatric cancers from adult-type solid tumors would be helpful to see if the trends in end-of-life care intensity hold true across both groups.

VERSION 2 – AUTHOR RESPONSE

Reviewer Comments	Response
Reviewer 1	

I have a major concern about the cohort included and the possibility of a time/age bias. Rather than following a cohort of those diagnosed with cancer < 25 years until they die or reach age 25 (only deaths under 25 are included), with censoring for those with incomplete follow-up, they instead include only those who are diagnosed with cancer < 25 years and also die before 2021. The abstract states that only deaths from 2012 are included, but this information is not given in the methods of the paper. We are not informed when records begin.<br><br>The structure of the cohort means that those towards the end (2021) are likely to be biased towards younger deaths. For example, suppose two children of the same age live for 3 and 5 years after diagnosis. If they are both diagnosed in 2010, they will both occur in the cohort, whereas if they are diagnosed in 2017, only the earlier death will be identified. Both will be within the denominator of the cohort, but only one death will be recorded. This corresponds with the pattern shown in table 1 of children who die at younger ages and later time points both having higher odds of high intensity treatment.	We thank the reviewer for the thorough review. We have responded where possible but also do not agree with all the comments<br><br>Apologies for the lack of clarity on the cohort construction. We include all **those who died** after a cancer diagnoses where the death occurred 2012-2022 and they were aged <35 years at the time of death. The child may have had a cancer diagnoses between 1/1/1988 to 31/12/2020. This has been added to page 5.<br><br>The example given by the reviewer of one child dying but both being in the denominator cohort is not correct therefore we do not agree that these are biased. In the given example, only one of the children is in the cohort at all (in the denominator of all included deaths and in the high intensity treatment group depending on treatment – the child who had not died by 31 December 2020 would not be included at all. Our cohort only included those who had died as are primary question was about end of life care.<br><br>If we had been answering different questions about the likely of death after specific treatment then a full cohort of all children diagnosed with cancer would have been more appropriate.
1. The abstract:<br>a) The aims as stated in the abstract (establish prevalence and assess the relationship with palliative care integration) do not appear to directly relate to the results as presented in the abstract.<br>b) The abstract results require some editing: the number of children studied should be stated; percentages should be presented with confidence intervals; how are 'health inequalities in treatments' defined?; how do the odds of dying in hospital in some subgroups relate to the stated aims?; what was the relationship between palliative care integration and high-intensity treatment (the second stated aim)?	a) we have made the aims clearer in the abstract<br>b) the numbers of children have been added. Confidence intervals are not necessary as these are full population data not a sample.<br>c) hospital deaths are the *most common* (as opposed to *the majority*) as all the other places of death are <45%

c) If the appropriateness of high-intensity care is hard to evaluate, what is the purpose of studying the prevalence and other aspects? As written, the abstract conclusions appear contradictory to the results (hospital deaths stated as most common, yet results state 45% hospital deaths).	
2. The summary section (iii) Implications for practice, theory and policy: How are these inferred from the study results presented?<br>a) The first statement seems to relate to something not studied here (advanced care plans, thorough discussion of care options) and just based on author belief.<br>b) Is non-hospital death preferred? What evidence is there for this? There may be an association between specialist palliative care integration and non-hospital death, but is this causally related in the inferred direction? (It may be that a non-hospital death is the causal driver of a better specialist care integration rating.) What evidence is there that comprehensive support will enable preferred place of death? How many children were not at a preferred place of death? (Was this asked within the study?)	Previous papers from this study have shown – these summary sections do not allow for references but these are provided in the discussion<br>a) we have amended the first statement "Providers should carefully assess appropriateness of high-intensity treatments and ensure a thorough discussion of care options with children and their families."<br>b) as stated and referenced in the discussion "Although most parents prefer their child to die at home, especially in cancer cases, we have little information from parents in minority ethnic groups.28<br>Previous qualitative data from the ENHANCE study has shown the lack of support results in less choice about place of care https://doi.org/10.1177/02692163251320204 and https://doi.org/10.1136/spcare-2025-005427<br>we did not have information on preferred place of death as these data are not currently collected I routine data– this was noted in implications for research
3. The secondary aim, stated at the end of the introduction, needs to clarify whether the aim is to determine the relationship between place of death and high-intensity cancer treatment, or place of death and palliative care integration.	This has been clarified "A secondary aim was assess the relationship between palliative care integration and place of death."
4. The supplementary appendix gives 3 additional survey questions, but it is unclear how these questions address non-response in the original.<br>Regarding the note below these new questions, the 'original response' to what question would be assumed to apply to the whole period, and how valid is this assumption? Why not use multiple imputation rather than a last	The revised survey detailed in the appendix contained *only* the three questions listed – these were not additional questions. The aim of this to reduce burden of response and elicit additional responses from those centres that had not responded to the original, longer survey (which had additional purposes as detailed in the relevant paper).<br>The new survey gave us additional information on when palliative care integration took place, so we preferred these data where available. In the absence of a response

observation carried forward approach (which can induce bias)?	to the new survey, we used the existing responses to the corresponding questions (now detailed in the appendix).<br><br>There doesn't appear to be a viable way of using multiple imputation to infer missing responses – it is not clear which data could be used. Respondents to the original survey do not have missing data for the question of interest; respondents to the new survey also do not; centres responding to neither survey provided us with no data on which an imputation model could be built.<br><br>We also address the potential impact of this in the supplement and in the main paper Discussion section – this assumption may lead to misclassification and a bias of results towards the null hypothesis of no association between integration of palliative care and the outcomes of interest
5. Do the authors use 'palliative care integration' and 'embeddedness of palliative care' interchangeably? Given the importance of this variable in the aims, there should be more detail of the construction of this variable and some evidence to support the reliability and validity of this. Reference 23 is given but no detail, and there is collapsing of this further to a binary outcome.	The construction of the variable is provided in the methods section<br><br>"Embeddedness of medical and nursing palliative care expertise and age-appropriate consult-led palliative care, were categorised as absent, partial or strong, as previously reported.[23] These were collapsed into a combined measure, 1 if there was strong embeddedness of palliative care across all three domains and 0 otherwise. " Embeddedness was used in the original survey we have added that the term integration of palliative care is used as more commonly used.
6. Figure S 12 requires more information. On what evidence was this constructed? Latent variables are not directly observable by definition, yet the grey variables shown here (defined latent) do not fit this criteria.	The DAG was constructed with reference to know associations from the literature and expected associations based on the expertise of the authors. Causal pathways were assumed unless there was a clear consensus that these were absent. The figure caption has been updated to refer to the grey variables as unobserved, rather than latent (the reviewer is correct that at least some of these are not intrinsically unobservable)
7. Figure S13 is reference in the paper, but is not given in the supplementary material.	Figure S13 is in the supplemental material
8. Age at diagnosis should be shown in figure 2.	added

9. Figure S2 referenced in text when Figure S1 is meant.	There was in fact no figure S1 and numbering started at S2. This has now been fixed, so the reference to S2 is correct now.
10. The text referring to Figure S2 (should be S1, Palliative Care paragraph of results), states that 6 centres reported full integration pre 2020. How is 6 obtained from the figure (it looks like 5)? Is 'full integration' here meant to correspond to 'strong embeddedness' described on page 6 in the methods?	6 is correct **by 2020** (as stated). Sheffield Children's had strong embeddedness **from the 2012-2015 period onwards** across all three domains. The other 5 had strong embeddedness across all three domains for the whole time period.
11. Where descriptive percentages are presented (eg 52% high intensity treatment), confidence intervals should be given.	These are whole population figures so confidence intervals are not required. We're not working on a sample of those who died.
12. Please clarify that the results shown in table1 have adjusted for within centre correlation. It is possible that there are within centre confounders which drive the differences in the outcomes. How can the authors separate the centre effect from the fact that palliative care integration is centre specific? This is another potential flaw in interpretation of the results.	As stated in the methods section (page 9 line 194) a random intercept model was used to account for within centre clustering.
13. At the start of the descriptive summary, the place of death is given for 92% of children. What about the other 8%?	This has been updated to specify that the other 8% were in 'other or unknown settings' and that the vast majority of these were 'unknown' in the cancer registry data – the few others were 'nursing home' or 'other'.
14. The choice of adjustment factors for the models presented in table 1 requires explanation. Why are some said to not require adjustment? It is likely that some of the factors are linked (for example some cancer categories being more common in some ethnic groups). Models could be used to investigate how these variables interact.	The DAG suggests that no adjustment is needed for these variables, with respect to the outcome. Taking the example of ethnic group, cancer type appears to be a mediator on the causal pathway from ethnic group to high intensity treatment (Figure S12) but it is not confounder. It is not appropriate to include mediators. The question being asked here is whether ethnic group influences prevalence of high intensity treatment. If it does, this may be due, in part, to different cancer types experienced (the question would remain – for another study - as to whether care is different for valid reasons due to the type of cancer).
Reviewer 2	
Very interesting/relevant paper describing a study with useful and direct implications for service	We thank the reviewer for their positive comments about this paper.

provision and commission policies/strategies.<br>The introduction is clear and justify the reasons to undertake the study. Although the topic is not original it is clearly explained the rational behind the study and how the results add to the current knowledge locally and in comparison to similar studies previously reported.<br>The material and methods sessions are easy to understand and the use of figure and diagrams help to follow the discussion.<br>Despite the acknowledgement of inability to use relative risk and need to use odds, I personally think that logistic regression is a simple and good choice to analyse this type of data. Sources and exemptions are nicely listed, making the study robust.	
The direct acyclic graph (DAG) in the appendix do not really add much to the data analysis.	The DAGs are essential to define the variables included in each of the regressions presented and so are included fo transparency. It is likely they will be of more relevance to those with an interest in the modelling approach than in the clinical implications.
Results are reported in a concise and logical manner and the discussion is relevant to the study aim. I found very appropriate the comparison with other countries and the two session on implications for research and for practice are excellent.<br>I particularly agree on the observation regarding need for further research into the place of death to understand if this is in accordance with patient and family advance care plan choices.<br>In the discussion the arguments are explained logically facilitating extrapolation of universal valid conclusions from the findings of the study.	
- line 292 change not chance<br>- line 308 extra "but"<br>- line 352 is missing the verb<br>- in the appendix line 31 ? in common	All these have been changed.<br><br>The % explanation has been added to figure 1 caption

- caption for fig 1 should also explained the percentages	
Reviewer 3	
This is important data for paediatric oncology services but the lack of ability to distinguish between palliative and curative chemotherapy is a limitation. Particularly as SACT data should include this information. Probably this is not completed and perhaps should be made mandatory to be able to identify when futile curative treatments are used close to end of life. It would be ideal of cause of death data was available too from death certification to identify deaths due to disease and deaths due to treatment.	These limitations were mentioned in the limitations section but we have added further detail on page 13 line 340<br><br>Noting that some of these data i.e. curative or palliative intent should be completed in the existing SACT dataset but were mainly missing.
