# Supplementary material for: High-intensity treatment is common at the end of life among children and young people who died of cancer: a retrospective cohort study
Source: BMJ Paediatr Open. 2026 Sep 1;10(1):e004575. doi: 10.1136/bmjpo-2026-004575 (PMC13536170; doi:10.1136/bmjpo-2026-004575)
Supplement: Supplementary file 2 [file bmjpo-10-1-s001.docx]

High intensity treatment at end-of-life for children and young people who died with cancer: a secondary data analysis in England

Stuart Jarvis^1^, Pandell Damun^2^, Sebastian Hinde^2^, Helen Weatherly^2^, Richard G. Feltbower^3^, Gayathri Subramanian^4^, Bob Phillips^5,6^ & Lorna K Fraser^7^

# Supplementary appendix

## Data linkage

For linkage to intensive care audit data, PICANet and ICNARC sent identifying information on children and young people aged under 25 years to NHS England. NHS England then returned identifiers for cohort members to these organisations, complete with a unique participant ID for each person, used by NHS England in their data provided to the research team. PICANet and ICNARC then also included this unique participant ID in their data provided to the research team. All data received by the research team were pseudonymised in this way.

## Details of derivation of outcomes data

#### Chemotherapy

The SACT, HES APC and HES outpatient datasets were searched for OPCS-4 codes[ref] indicating IV chemotherapy (delivery codes X721 or X352). In SACT, searches were also made for administration route 1 and no recording of “NOT CHEMO” in the drug group field.

Oral chemotherapy events were identified in SACT using records with administration route 2 recorded (again in the absence of “NOT CHEMO” in the drug group field).

#### Radiotherapy

Radiotherapy was determined from presence of records in the RTDS. These were classified as palliative when indicated as such in the data (radiotherapyintent field = 1).

#### Hematopoietic stem cell transplantation

Hematopoietic stem cell transplantation (HSCT) was determined from the ICU data (in PICANet data there is a specific flag for HSCT) and from HES APC data using costing codes for bone marrow or stem cell transplantation (HRGs SA19-23 and SA26-40) and OPCS-4 codes for the relevant procedures (codes starting W34).

#### Mechanical ventilation

Mechanical ventilation was determined from flags in the ICU data (ICNARC: advancedventilation field; PICANET: invventet or invventtt).

#### Emergency department visits

Emergency department visits were determined from presence in the HES A&E dataset. Date of visit was recorded to determine proximity to death

#### Hospital admissions

Hospital admissions were determined from presence in the HES APC dataset. Date of visit was recorded to determine proximity to death. Individual records, each indicating finished consultant episodes of care, were combined into admissions so that distinct admissions could be counted.

#### ICU admissions

ICU admissions were determined from presence in PICANet or ICNARC datasets. Dates of admission were used to determine proximity to death.

## Survey - integration of palliative care in main treatment centres

The original survey has been previously reported as detailed in the main text. The updated survey contained only three questions (Figure S1).

Figure S 1: Questions included in the revised survey.

The previous survey did not ask when services were introduced; where centres did not respond to the new survey, their response to the corresponding original survey questions (as detailed in the ‘Embeddedness of palliative care’ section of table 1in Bedendo et al, 2025 as referenced in the main text) was assumed to apply to the whole of the 2012-2020 period as it was not known when services were introduced This may have the effect of including, in the group considered to have well integrated palliative care services, centres where this was not the case for some of the period. This may bias results based on this towards the null hypothesis of no difference in intensity of end-of-life treatment between centres with and without palliative care integration.

Figure S 2: Summary of medical palliative care (PC) integration, nurse PC integration and age-appropriate PC team integration at each of the main treatment centres, based on survey responses. No responses were received from Leicester or Manchester; the response from GOSH was not sufficiently complete to assess PC integration.

## Components of the main measure of high intensity treatment at end of life

#### Components of high intensity treatment

The composite outcome of receiving HIT at the end of life was dominated by two components: multiple hospitalisations within the last 30 days (present for 39% of cohort members) and any ICU admission within the last 30 days (20%), with very few having multiple ED visits (4%) or receiving IV chemotherapy (6%) within the last 14 days of life.

The prevalence of multiple hospitalisations in the last 30 days of life varied little across demographic characteristics, except it was lower for under 1s (possibly due to being in hospital and not being at risk of multiple admissions) (figure S2, appendix). There were variations by cancer type, with highest prevalence for leukaemia and neuroblastoma and lowest for central nervous system and other cancers.

The prevalence of ICU admission within the last 30 days of life varied more, being most common for those who died aged 0-3 years (figure S3, appendix). ICU admissions in the last 30 days increased over time. It was most common for children and young people with leukaemia and lymphoma. There were also variations by main treatment centre, with ICU admissions most common at Manchester, Alder Hey, GOSH and Birmingham.

Intravenous chemotherapy in the last 14 days of life increased over time but showed no substantial difference by demographics (figure S1, appendix). Those with leukaemia or lymphoma were most likely to have IV chemotherapy in the last 14 days of life

Emergency Department visits were infrequent in children but became more common in later teenage years and for young people aged 20-24 years at death (figure S4, appendix). ED visits were most prevalent for those with renal hepatic, germ cell or skin cancers, possibly reflecting the distribution of cancers in the 20-24 year age group. ED visits were more prevalent for the Black and South Asian ethnic groups and for those from the most deprived areas. There were substantial variations by main treatment centre, likely reflecting differences in policy on ward access and ED visits were less common at end of life in centres with full integration of palliative care.

#### Secondary measures of treatment intensity

14% of the cohort received mechanical ventilation within the last 14 days of life. This was most common in those who died youngest (0-3 years) (figure S9, appendix). Those with leukaemia or lymphoma most commonly received mechanical ventilation and prevalence increased over time.

6.5% received radiotherapy within the last 30 days of life (2.6% within the last 14 days). Radiotherapy was most common in older age groups (figure S6, appendix). It was most common for those with lymphoma, neuroblastoma, bone, soft tissue or skin cancers. Excluding those marked as receiving radiotherapy only with palliative intent, 2.5% received radiotherapy within the last 30 days of life (1.0% within the last 14 days) and this appeared to have decreased over time, although this could be due to increased recording of palliative intent (figure S7, appendix).

The lack of a HSCT code in ICNARC data meant that stem cell transplantation was rarely detected in those aged over 16 years at death (figure S8, appendix). HSCT was most commonly seen in the youngest (0-2 years) at death and increased over time towards the end of the study period, from 5.3% in 2016 to 11.6% in 2020. It was most common for those with leukaemia, lymphomas, or neuroblastoma. There were substantial variations by main treatment centre (only some centres will routinely perform transplants).

Oral or intravenous chemotherapy was received by 7.1% of the cohort within the last 14 days of life. In common with intravenous chemotherapy, it increased over time (figure S5, appendix). Those with leukaemia or lymphoma were most likely to have oral or intravenous chemotherapy in the last 14 days of life.

Figure S 3: Prevalence of multiple hospital admissions within the last 30 days of life.

Figure S 4: Prevalence of intensive care unit admission within the last 30 days of life.

Figure S 5: Prevalence of intravenous chemotherapy within the last fourteen days of life.

Figure S 6: Prevalence of multiple Emergency Department visits within the last 30 days of life

## Secondary measures of high intensity treatment at end of life

Figure S 7: Prevalence of mechanical ventilation in the last 14 days of life.

Figure S 8: Prevalence of radiotherapy in last 30 days of life.

Figure S 9: Prevalence of radiotherapy, not marked as having palliative intent, in last 30 days of life.

Figure S 10: Prevalence of haematopoietic stem cell transplantation in the last 100 days of life (note: data limitations for young people aged >16 years lead to an underestimate for these ages, as noted in the main text).

Figure S 11: Prevalence of oral or intravenous chemotherapy in the last 14 days of life.

## Directed acyclic graphs used in model development

### High intensity treatment at end of life

Figure S 12: Directed acyclic graph (DAG) for hypothesised relationships between high-intensity treatment at end-of-life (outcome, blue, black outline), the exposure of interest (integration of palliative care, green), adjusted-for variables (white) and unobserved variables (grey). The same DAG was used to define models for other exposures of interest, with adjusted-for variables changed as necessary.

### Hospital as place of death

Figure S 13: Directed acyclic graph (DAG) for hypothesised relationships between hospital as place of death (outcome, blue with black outline), the exposure of interest (integration of palliative care, green), adjusted-for variables (white), unadjusted for observed variables (blue, blue outline) and unobserved variables (grey). The same DAG was used to define models for other exposures of interest, with adjusted-for variables changed as necessary.

## Time from Diagnoses to Death

Tabel S1- High Intensity treatment by time from diagnoses to death.

| Diagnosis to death | Deaths aged >= 5 years | Deaths aged >= 5 years | All deaths | All deaths |
| --- | --- | --- | --- | --- |
|  | % with HIT | N with HIT | % with HIT | N with HIT |
| < 90 days | 61 | 389 | 59 | 236 |
| 90-180 days | 55 | 185 | 52 | 129 |
| 180-365 days | 56 | 461 | 53 | 330 |
| 1-2 years | 51 | 541 | 48 | 427 |
| 2-5 years | 47 | 438 | 45 | 393 |
| > 5 years | 45 | 204 | 45 | 204 |

## Sensitivity analysis – regression models excluding 2020

Table S2: Multivariable logistic regression model results for risk of high-intensity treatment at end-of-life: senstitivty analysis covering only deaths in the period 2012-2019 inclusive.

|  | **Outcome: high intensity treatment at end of life** | | **Outcome: hospital as place of death** | |
| --- | --- | --- | --- | --- |
|  | **Odds ratio** | **95% CI** | **Odds Ratio** | **95%CI** |
| **Specialist paediatric palliative care integration** (ref: incomplete integration) [1] |  |  |  |  |
| Full integration | 0.92 | 0.78-1.08 | 0.60 | 0.39-0.94 |
| **Age at death** (ref: 5-10 years) [2] |  |  |  |  |
| Under 1 year | 1.67 | 1.11-2.52 | 3.15 | 2.04-4.86 |
| 1 to 4 years | 2.42 | 1.9-3.08 | 1.66 | 1.31-2.11 |
| 11-15 years | 0.82 | 0.65-1.02 | 1.24 | 0.98-1.57 |
| 16-17 years | 1.08 | 0.82-1.41 | 1.66 | 1.25-2.2 |
| 18-25 years | 0.94 | 0.78-1.13 | 1.97 | 1.62-2.4 |
| **Sex** (ref: male) [3] |  |  |  |  |
| Female | 0.92 | 0.80-1.05 | 1.01 | 0.88-1.16 |
| **Ethnic group** (ref: White) [3] |  |  |  |  |
| Black | 1.06 | 0.77-1.45 | 2.33 | 1.68-3.24 |
| South Asian | 1.05 | 0.81-1.36 | 1.92 | 1.48-2.5 |
| Mixed or other | 1.03 | 0.82-1.30 | 1.87 | 1.48-2.37 |
| **Deprivation category** (ref: 1, most deprived) [4] |  |  |  |  |
| 2 | 1.03 | 0.84-1.27 | 0.78 | 0.63-0.97 |
| 3 | 0.95 | 0.77-1.17 | 0.68 | 0.55-0.85 |
| 4 | 0.86 | 0.69-1.06 | 0.69 | 0.55-0.86 |
| 5, least deprived | 0.95 | 0.76-1.18 | 0.64 | 0.51-0.81 |
| **Cancer category** (ref: solid tumours) [5] |  |  |  |  |
| Haematological | 2.65 | 2.2-3.18 | 3.46 | 2.88-4.16 |
| Central Nervous System | 0.44 | 0.36-0.52 | 0.74 | 0.61-0.89 |
| **Year of death** (per year) [3] | 1.03 | 1.00-1.06 | 1.04 | 1.01-1.07 |

[1] Adjusted for age at death, sex, ethnic group, deprivation category, cancer category, and year of death

[2] Adjusted for year of death

[3] Unadjusted

[4] Adjusted for age at death, sex, ethnic group, and year of death

[5] Adjusted for age at death, sex, ethnic group, deprivation category, and year of death

Table S 3: Multivariable logistic regression model results for risk of high-intensity treatment at end-of-life: senstitivty analysis separted by age at diagnoses ( <18 years and 18 years +).

|  | **Outcome: high intensity treatment at end of life – diagnosis under 18** | | **Outcome: high intensity treatment at end of life – diagnosis 18+** | |
| --- | --- | --- | --- | --- |
|  | **Odds ratio** | **95% CI** | **Odds Ratio** | **95%CI** |
| **Specialist paediatric palliative care integration** (ref: incomplete integration) [1] |  |  |  |  |
| Full integration | 0.92 | 0.76-1.12 | 0.7 | 0.51-0.96 |
| **Age at death** (ref: 5-10 years) [2] |  |  |  |  |
| Under 1 year | 1.56 | 1.06-2.3 |  |  |
| 1 to 4 years | 2.32 | 1.84-2.92 |  |  |
| 11-15 years | 0.82 | 0.66-1.01 |  |  |
| 16-17 years | 1.07 | 0.83-1.39 |  |  |
| 18-25 years | 0.69 | 0.54-0.87 |  |  |
| **Sex** (ref: male) [3] |  |  |  |  |
| Female | 0.93 | 0.8-1.08 | 0.89 | 0.69-1.15 |
| **Ethnic group** (ref: White) [3] |  |  |  |  |
| Black | 1.01 | 0.73-1.42 | 0.97 | 0.5-1.87 |
| South Asian | 1.07 | 0.81-1.42 | 1.14 | 0.68-1.92 |
| Mixed or other | 1.06 | 0.82-1.36 | 0.95 | 0.61-1.47 |
| **Deprivation category** (ref: 1, most deprived) [4] |  |  |  |  |
| 2 | 0.96 | 0.76-1.2 | 1.19 | 0.82-1.72 |
| 3 | 0.99 | 0.79-1.25 | 1.15 | 0.78-1.7 |
| 4 | 0.74 | 0.58-0.94 | 1.23 | 0.82-1.85 |
| 5, least deprived | 0.88 | 0.69-1.13 | 1.49 | 0.97-2.29 |
| **Cancer category** (ref: solid tumours) [5] |  |  |  |  |
| Haematological | 2.85 | 2.31-3.51 | 2.48 | 1.8-3.44 |
| Central Nervous System | 0.49 | 0.41-0.6 | 0.28 | 0.17-0.44 |
| **Year of death** (per year) [3] | 1.02 | 0.99-1.06 | 1.06 | 1-1.13 |

[1] Adjusted for age at death, sex, ethnic group, deprivation category, cancer category, and year of death

[2] Adjusted for year of death

[3] No adjustment needed

[4] Adjusted for age at death, sex, ethnic group, and year of death

[5] Adjusted for age at death, sex, ethnic group, deprivation category, and year of death
